# Predicting quantitative traits from genome and phenome with near perfect accuracy

**DOI:** 10.1038/ncomms11512

**Published:** 2016-05-10

**Authors:** Kaspar Märtens, Johan Hallin, Jonas Warringer, Gianni Liti, Leopold Parts

**Affiliations:** 1 Institute of Computer Science, University of Tartu, Tartu 50409, Estonia; 2 Institute for Research on Cancer and Aging, University of Sophia Antipolis, Nice 02 06107, France; 3 Department of Chemistry and Molecular Biology, Gothenburg University, Gothenburg 40530, Sweden; 4 Centre for Integrative Genetics (CIGENE), Department of Animal and Aquacultural Sciences, Norwegian University of Life Sciences, Ås N-1432, Norway; 5 Wellcome Trust Sanger Institute, Wellcome Trust Genome Campus, Hinxton CB101SA, UK

## Abstract

In spite of decades of linkage and association studies and its potential impact on human health, reliable prediction of an individual's risk for heritable disease remains difficult. Large numbers of mapped loci do not explain substantial fractions of heritable variation, leaving an open question of whether accurate complex trait predictions can be achieved in practice. Here, we use a genome sequenced population of ∼7,000 yeast strains of high but varying relatedness, and predict growth traits from family information, effects of segregating genetic variants and growth in other environments with an average coefficient of determination *R*^2^ of 0.91. This accuracy exceeds narrow-sense heritability, approaches limits imposed by measurement repeatability and is higher than achieved with a single assay in the laboratory. Our results prove that very accurate prediction of complex traits is possible, and suggest that additional data from families rather than reference cohorts may be more useful for this purpose.

## Abstract (web summary)

Heritability of complex traits can be finely dissected using yeast crosses. Here, Kaspar Märtens and colleagues show that quantitative traits in yeast can be predicted from genotype and phenotype data with an average coefficient of determination of 0.91, providing the most accurate predictions of complex traits to date.

## Graphical abstract

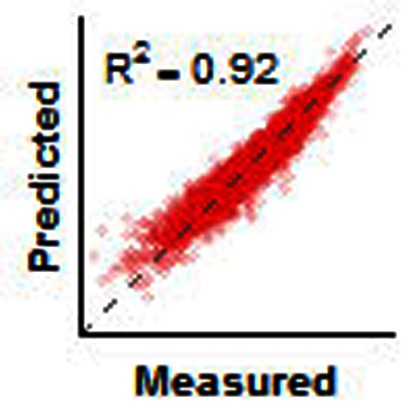

Disease incidence can be predicted based on the health record^1^, the family history^2^ or the genetic risk due to predisposing genetic variants segregating in the population^3^. Each of these sources of information carries signal about the trait, but is not sufficient for accurate prediction^2^,^4^,^5^. For example, the genetic variants mapped to a trait in genome-wide association studies do not estimate disease risk well, with the vast majority of the heritable variation not accounted for^6^,^7^. Even with very large numbers of mapped alleles^8^, purely genomic prediction accuracies still lag far behind narrow sense heritability estimates^9^.

An important question of whether this is due to paucity of data, or perhaps more fundamental limitations, can be attacked by predicting phenotypes in model organisms^10^,^11^. In particular, crosses of founders in the yeast system have circumvented many of the technical difficulties associated with human genetic analyses, and illuminated genetic basis of variation in molecular traits^12^,^13^,^14^, cellular phenotypes^15^,^16^,^17^, missing heritability^18^ and role of interactions^19^,^20^,^21^. Genome-based prediction has successfully explained most of the trait variation in two organism phenotypes using up to five mapped alleles^20^,^22^, and approached narrow-sense heritability accuracy in a large-scale cross^18^. For yeast, growth in various environments is an analogue of the health record, family history is approximated by phenotypes of closely related individuals, and risk variants can be mapped as for humans. Thus, we can test whether accurate phenotype prediction for more complex traits is possible in practice, and what the constraints are.

Here, we use a recent resource of over 7,000 diploid hybrid yeast strains of high relatedness^23^ to predict their growth phenotypes. Combining genetic and phenotypic data in a linear mixed model (LMM) framework, as well as using a recently introduced mixed random forest (MRF) approach, we predict growth traits with accuracies above their narrow-sense heritability, and approaching limits set by measurement repeatability. We find that both relatedness and variant-based predictions are greatly aided by availability of very close relatives, whereas information from a large number of more distant relatives fail to improve predictive performance when closer relatives are included. Our results suggest that prediction is improved by both data from closer relatives that share much of the genome, as well as additional phenotype measurements that can capture aspects of unique environment and effects too small to be detected by mapping.

## Results

### Study population

**Figure 1:**
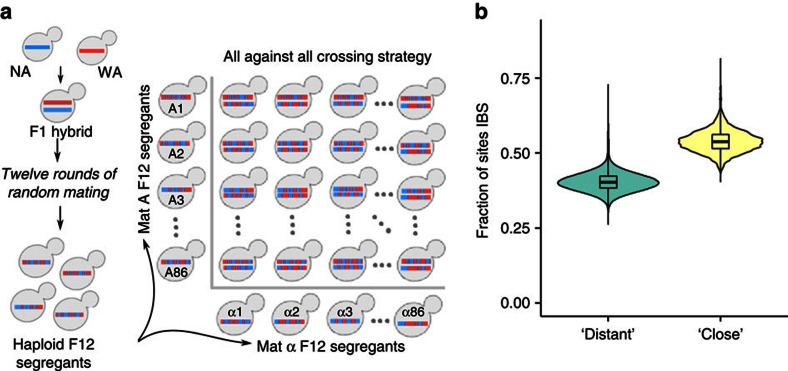
Experiment population. The 7,396 studied individuals are diploid hybrids that were constructed by systematic mating of 86 F12 *MATa* haploid yeast segregants to 86 *MATα* individuals, in all pairwise combinations. (**a**) Two-stage crossing scheme, starting from the West African (WA) and North American (NA) parents gives a large, diverse, diploid population. (**b**) Distribution of fraction of sites with identical genotype for pairs of hybrids is bimodal. The frequency of individual pairs that are identical by genotype state (IBS) at fraction f of the sites (y-axis) is different for pairs that share one parent (‘close', right), and ones that do not (‘distant', left).

We made use of 7,396 diploid hybrid *Saccharomyces cerevisiae* strains with phased whole-genome sequences from the collection of diploid phased outbred lines^23^. Owing to the two-stage crossing scheme (Fig. 1a), each of these hybrids has 170 relatives that share one chromosome in every chromosome pair (expected fraction of segregating site genotypes identical by state *f*=0.5), and 7,225 ones for which no complete chromosome is shared, but a substantial part of linkage blocks and allele combinations are (expected *f*=0.375, Fig. 1b). We refer to these levels of relatedness as ‘close' and ‘distant', respectively, noting that both classes correspond to close kinship. After filtering out individuals with aneuploidies and contamination, we retained 6,642 strains for analysis. Population growth of individual diploid hybrids was measured^24^ in nine environments in technical and biological duplicate, growth estimates were normalized against hundreds of densely spaced internal standards and the replicate average was used for analysis. The environments challenge different cellular functions, covering energy sources (for example, galactose), osmotic stress (for example, NaCl) and cancer drugs (for example, rapamycin, Supplementary Table 1). As reported before^23^, the phenotype means have large narrow-sense heritabilities (*h*^2^) and repeatabilities (*H*^2^, broad-sense heritability; median *h*^2^=80%, *H*^2^=94%, standard error=0.09, Supplementary Tables 2 and 3), and the traits are not independent (pairwise Pearson's *r*^2^=0.01–0.49, Supplementary Fig. 1), reflecting shared genetic, epigenetic and environmental influences (Supplementary Fig. 2).

### Accurate genome-aided phenotype prediction

**Figure 2:**
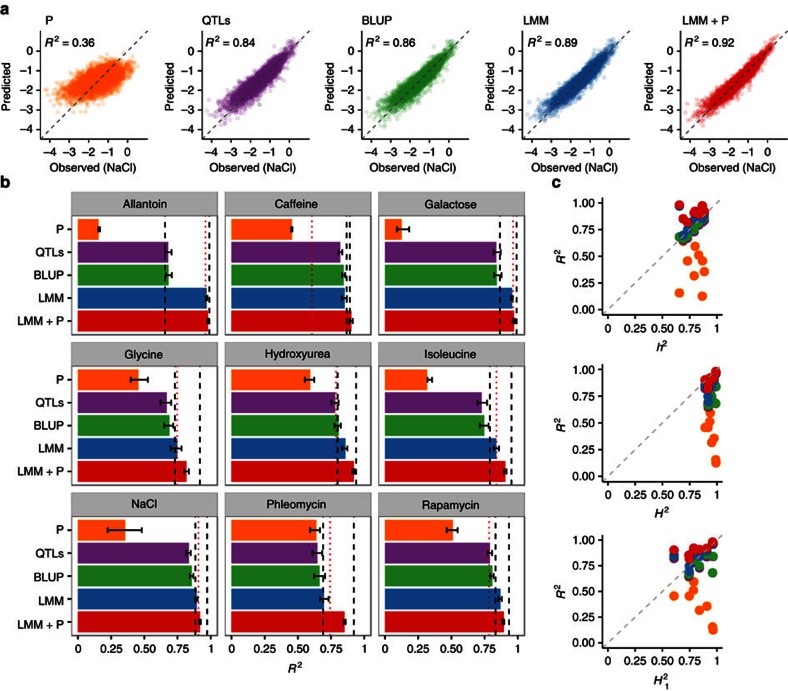
Prediction accuracy. All panels contain five model classes: linear regression on other phenotypes (‘P', yellow), linear regression with additive effects determined by forward selection (‘QTLs', purple), prediction based on the realized genetic relatedness (‘BLUP', green), the best LMM with additive and interaction effects (‘LMM', blue) and the best LMM with additive and interaction effects together with other phenotypes (‘LMM+P', red). All prediction accuracies denote coefficient of determination *R*^2^, and are determined by fourfold cross-validation. (**a**) Models using a single source of information predict less accurately than a combined one. Predicted (*y* axis) and observed (*x* axis) growth in NaCl for every measured hybrid strain (dots) for each model class, with coefficient of determination (*R*^2^) of the predictions labelled. Perfect predictions would lie on the grey dashed line *y*=*x*. (**b**) Linear mixed models with information from other phenotypes give very accurate predictions. Predictive performance (*R*^2^, *x* axis) for different models (*y* axis) for each of the measured phenotypes (nine boxes). Bars indicate the range of *R*^2^ over the four cross-validation folds. The dashed lines show narrow-sense heritability *h*^2^ (black, left) and repeatability *H*^2^ (black, right) estimates for the mean phenotype, and the dotted line (red) shows repeatability of a single measurement *H*_1_^2^. (**c**) Prediction can be more accurate than one measurement. Prediction accuracy of mean phenotype (*R*^2^, *y* axis) compared with different types of heritability estimates (*x* axis) for the four model classes: narrow-sense heritability of average phenotype (*h*^2^, top panel), repeatability of average phenotype (*H*^2^, middle panel) and repeatability of a single measurement (*H*_1_^2^, bottom panel). Grey dashed lines denote the identity *y*=*x*.

We first tested how well different genomic and phenomic data predicted growth phenotypes in our population (Fig. 2a and Supplementary Fig. 3), and then combined them using LMMs^25^. We obtained predictions via fourfold cross-validation, with the training set randomly sampled from both close and distant relatives (Methods). One growth trait could be predicted from the rest with reasonable accuracy (Fig. 2b ‘P', median *R*^2^=0.48), and the quality of prediction depends on the strength of pairwise correlations of the phenotypes. The genomic best linear unbiased predictor (BLUP), an additive model based on realized genetic relatedness alone, captures the pedigree structure in the population, and achieves prediction accuracies very close to the narrow-sense heritability estimates (Fig. 2b ‘BLUP', median *R*^2^=0.77, 98% of *h*^2^ explained). These predictions are near-identical to a simple midparent approach (Pearson's *r*^2^>0.99, Supplementary Fig. 4). Thus, the genetic similarity between individuals explains nearly all additively heritable variation in our population.

Next, we mapped quantitative trait loci (QTLs) in each environment, and asked how well they predict growth in that environment. A small number of single nucleotide polymorphisms (SNPs) with the largest effects explain a sizeable portion of additive variance, but for all traits the prediction accuracy remains lower than BLUP's (for example, median *R*^2^=0.58 versus 0.81 for 10 QTLs, Supplementary Fig. 5). When up to 50 SNPs are included in the model, the accuracy reaches *h*^2^ (Fig. 2b, ‘QTLs', median *R*^2^=0.78, 98% of *h*^2^ explained), with predictions very similar to BLUP (*r*^2^>0.97, Supplementary Fig. 6). Therefore, all tested methods that consider additive genetic effects reach the same, near-*h*^2^ performance, and there is no missing narrow-sense heritability in our experiment. Extending to the LMM framework to include genetic background, dominance and interaction effects gave a modest further improvement (median increase of *R*^2^ by 0.06), mainly due to dominance effects of strongest QTLs for allantoin and galactose (Fig. 2b, ‘LMM', median *R*^2^=0.86).

We then included other phenotypes measured for the same individual as covariates in the model, and achieved median prediction accuracy of 0.91 (Fig. 2b ‘LMM+P'). To our knowledge, this is the highest for complex traits to date^26^,^27^, exceeding narrow-sense heritability for all nine phenotypes and approaching repeatability (Fig. 2c, 96% of *H*^2^ explained). For each of the measured traits, our predictions of the mean phenotype (that is, the average of four replicate measurements) have lower error than a single growth experiment (Fig. 2c). The combined model improves over others especially when a large proportion of heritable non-additive variation is not captured by interaction and dominance effects (Supplementary Fig. 2).

### Predictions based on closer relatives are more accurate

**Figure 3:**
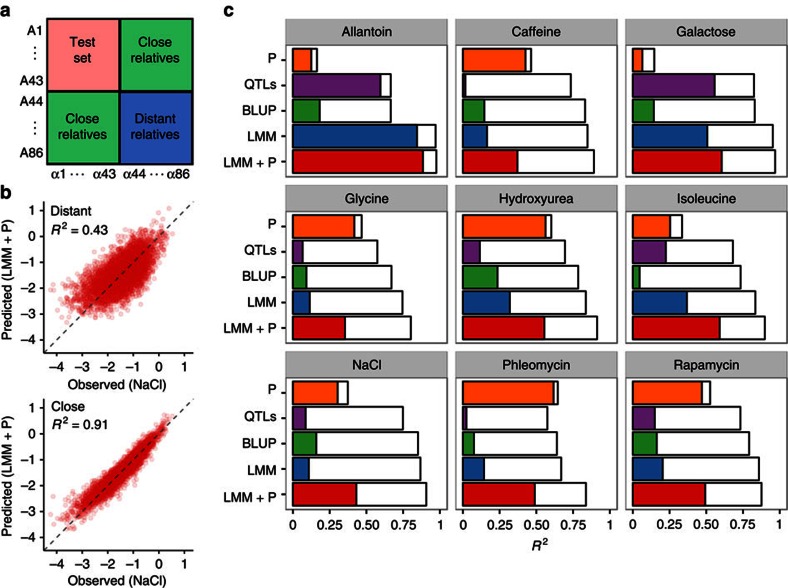
Close relatives improve predictions. (**a**) To cover two training scenarios, that is, fitting models on ‘close' (expected fraction of sites identical in genotype *f*=0.5) or ‘distant' (expected *f*=0.375) relatives, we partitioned all individuals into four equally sized groups. For a fixed test set (red box), we distinguish between training on close relatives (individuals who have a common parent with one test set individual, green box) and more distant relatives (no common parents with any test individual, blue box). As the number of close relatives is twice the number of distant relatives, we downsampled the former. Predictions are obtained by fourfold cross-validation. (**b**) Close relatives greatly contribute to genome-based prediction accuracy. Predicted (*y* axis) and observed (*x* axis) growth for test set individuals (red dots) in NaCl using the best LMM+P model in ‘distant' (top) and ‘close' (bottom) training scenarios. Grey dashed line denotes the identity *y*=*x*; coefficient of determination *R*^*2*^ is labelled on the plot. (**c**) Distant relatives are more difficult to predict in each environment. Predictive performance (*R*^2^, *x* axis) of different model classes (*y* axis) in two training scenarios: ‘Distant' (colored bars) and ‘Close' (white bars) for each of the nine environments (boxes).

**Figure 4:**
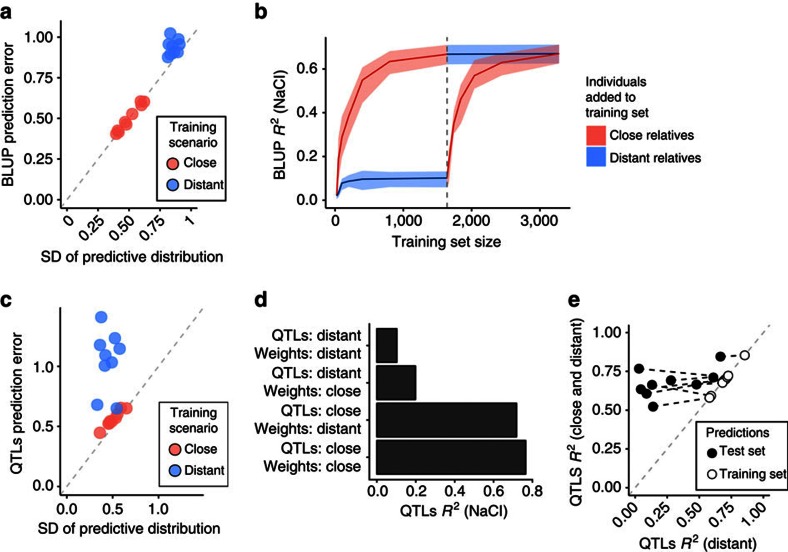
Causes of improved prediction performance for close relatives. (**a**) BLUP predictions from distant relatives are less accurate because of a more uncertain model-derived predictive distribution. Prediction error (*y* axis, standard deviation of the residuals) compared with the standard deviation of the predictive distribution (*x* axis) for the nine environments, when trained on distant (blue dots) or close relatives (red dots). (**b**) BLUP predictions are more accurate when the model is trained on a small number of close relatives compared with a large set of distant relatives. Predictive performance of BLUP (*R*^2^, *y* axis) improves with expanding the training set (size on *x* axis) with individuals closely (red line) or distantly (blue line) related to the test set. From the dashed grey line onwards, distant relatives are added to the training set of closely related individuals, and vice versa. Shaded regions denote the range of *R*^2^ over the four cross-validation folds. (**c**) Unlike for BLUP in **a**, the less accurate predictions from the QTL model in the ‘Distant' training scenario are not in accordance with uncertainty in the model-based predictive distribution. (**d**) Low QTL predictive ability for out-of-sample distant relatives is mainly due to discrepancies between the sets of mapped QTLs, not their estimated effects. Predictive performance (*R*^2^, *x* axis) of the QTLs model, stratified by training sets used for QTL mapping (model selection) and weight estimation (model fitting). QTL mapping and weight estimation are carried out under four training scenarios (*y* axis): both stages in distant relatives (‘QTLs: Distant, Weights: Distant'), both in close relatives (‘QTLs: Close, Weights: Close'), QTLs mapped in distant relatives and weights estimated in close relatives (‘QTLs: Distant, Weights: Close'), or vice versa (‘QTLs: Distant, Weights: Close'). (**e**) A minor change in the training set (replacing 1% of distant relatives with close ones) has a profound effect on out-of-sample QTL-based prediction accuracy. Out-of-sample (black dots) and in-sample (white dots) predictive performance (*R*^2^) of QTLs model in two scenarios: trained on distant relatives only (*x* axis) or when 1% is replaced with close relatives (*y* axis).

So far, our predictions for each test individual were obtained from models that were trained with data from its close relatives that share half of the complete chromosomes. We observed that errors were larger when close relatives were not available (for example, Fig. 3b and Supplementary Fig. 7). Thus, we next compared two training scenarios—‘close relatives', where each member of the test set has several close relatives in the training set (expected fraction of identical site genotypes *f*=0.5), and ‘distant relatives', where test set individuals are not as closely related to anyone in the training set (expected *f*=0.375, Fig. 3a). When training on close relatives, predictions based on other traits of the same individual are slightly more accurate (median improvement=0.04, Fig. 3c, ‘P'), whereas BLUP performs substantially better. On average, BLUP achieves *R*^2^ of 0.14 when trained on distant relatives and 0.76 on close ones (Fig. 3c, ‘BLUP'). This difference is explained by the larger uncertainty of the predictive distribution based on distant relatives: the observed errors are near-perfectly calibrated to their model-derived standard errors (Fig. 4a, *r*^2^=0.96). Accuracy increases markedly even with a small number of close relatives included in the training data, whereas adding more distant relatives to close ones does not improve predictions (Fig. 4b, Supplementary Fig. 8). For example, adding on average just five close relatives per test individual rises the median *R*^2^ from 0.15 to 0.65, but complementing the training set of close relatives by all distant relatives has a negligible effect (median *R*^2^=0.79 versus 0.81).

Perhaps surprisingly, training on close relatives also improved QTL-based predictions. For near-monogenic traits (for example, growth in allantoin and galactose), the accuracies were similar for both training scenarios (Fig. 3c ‘QTLs'). However, for more complex traits, the QTL model trained on distant relatives reaches high accuracy in the training data, but does not perform well out of sample, with 61% median decrease in accuracy (respective decrease for close relatives is 3%, Fig. 4e). In this case, the prediction uncertainties are similar (Fig. 4c), and most of this difference is explained by model selection. When we mapped QTLs in close relatives, but estimated their weights on distant relatives, the prediction accuracy decreased from 0.73 to 0.65 compared with carrying out both procedures on close relatives (Fig. 4d and Supplementary Fig. 9). Conversely, mapping QTLs in distant relatives and fitting their weights in close relatives resulted in a much lower *R*^2^ of 0.31. Including close relatives in training gives a more faithful approximation of the phenotypic covariance structure (Supplementary Fig. 10), which explains the large gap between out-of-sample and in-sample performance for distant relatives (Fig. 4e). Notably, prediction accuracy drops substantially, even when just 1% of the training data changes (Fig. 4e, filled versus empty markers).

Combining genomic and phenotypic information (LMM+P) to predict from distant relatives gives accuracies similar to combining QTLs and phenotypic information. For traits where genomic prediction on distant relatives does not work well (for example, caffeine, glycine, phleomycin), this model performs similarly to using other phenotypes only or even slightly worse (median improvement 0.02, Fig. 3c ‘LMM+P'). However, for traits with large effect QTLs (allantoin, galactose, isoleucine), genetic information helps prediction even if BLUP is not accurate.

### Prediction performance is consistent for alternative models

**Figure 5:**
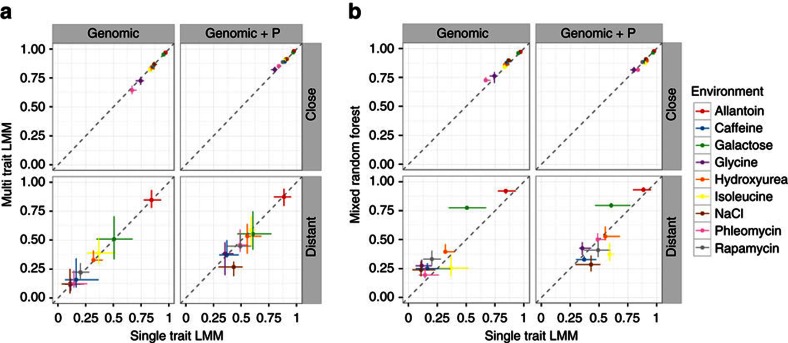
Prediction performance is similar for a range of model classes. Prediction performances of additional published methods to standard linear mixed models (LMMs), both on close and distant relatives. All results are shown for two training scenarios (close and distant relatives, panels ‘close' (top) and ‘distant' (bottom)) and two types of prediction: purely genomic prediction (panel ‘genomic', left), and combined genomic and phenomic prediction (panel ‘genomic+P', right). Both *x* and *y* axes represent the coefficient of determination *R*^2^, and the horizontal and vertical error bars denote the range of *R*^2^ over four cross-validation folds. (**a**) Multi-trait linear mixed models (MT-LMMs) perform similar to single-trait LMMs. Predictive performance (*R*^2^) for each environment (dots with various colours) for single-trait models (*x* axis) and multi-trait models (*y* axis). (**b**) Mixed random forests (MRFs) perform similar to single-trait LMMs. Predictive performance (*R*^2^) for single-trait LMMs (*x* axis) and MRFs (*y* axis).

Other methods for genome-aided trait prediction have either included other phenotypes directly in the model or are compatible with doing so^25^,^28^,^29^. We confirmed that these prediction implementations give results that are concordant with ours. First, we tested the multi-trait LMM (MT-LMM) that jointly infers the effects of genotype and other phenotypes^25^. This method gave results nearly identical to the LMM+P approach on both close and distant relatives, in which we first regressed the effect of phenotypes, and then fit a genomic model on the residuals (Fig. 5a). Second, we applied the recently published MRF, which accounts for population structure and captures nonlinear genetic effects^28^, and can use the other measured phenotypes as predictors. This method also performed similar to the combined LMM (median *R*^2^ 0.91 versus 0.91) for close relatives, with no consistent difference across the traits (Fig. 5b, top row). For distant relatives, the MRF had more accurate pure genomic predictions than a LMM for 8 of 9 traits, and when including phenotype information for both models, 4 of 9 traits (Fig. 5b, bottom row).

## Discussion

We predicted nine heritable traits in a population of 6,642 yeast strains of varying high relatedness, and achieved accuracies over 90%, very near the repeatability limit. To our knowledge, these are the most precise out-of-sample predictions of complex traits to date. There is almost no missing narrow- or broad-sense heritability, proving that very accurate genome-aided predictions can be obtained in practice, in contrast to relatively poor genomic prediction performance for human cohorts, for example, *R*^2^<0.16 using unrelated individuals, and <0.37 for close relatives^9^. Our predictions outperformed the traditional mid-parent approach that is limited to narrow-sense heritability, but has been predicted to remain unsurpassed in accuracy for humans^30^.

The improvement in predictive ability using phenotype data is due to capturing additional signal from the non-additive genetic and environmental components, reflecting the extent to which these are shared between the traits. Their relative contribution can somewhat be gauged from the additional accuracy of the LMM+P model over the standard LMM that accounts for mapped additive, dominance and interaction effects. The improvement is largest for traits that have a large gap between narrow and broad-sense heritabilities (phleomycin, hydroxyurea, glycine, isoleucine), which is not caused by a single dominant allele (galactose, allantoin). Any remaining difference is potentially due to both weak interaction and dominance effects not included in the LMM during model selection. Standardization, distribution of replicates across multiple pre-culture and experimental batches, and normalization of phenotypes to very densely spaced internal controls are expected to minimize the influence of shared environmental variation across plates^24^. A small contribution of shared environment is consistent with the phenotypic covariance decomposition (Supplementary Fig. 2), and sizes of variance components due to the 2nd and 3rd order interactions that are difficult to map^23^,^31^. Although we cannot completely exclude that a small fraction of the phenotype covariance reflects shared environmental variation, for example, in the form of nutrient access, initial population size or exposure to stress, the residual covariance has been empirically demonstrated to be smaller than our prediction improvements for most traits^24^. Regardless, additional measured phenotypes from the individual can clearly inform on all these sources of variation, circumventing the need to explicitly ascertain their effects.

Genomic prediction methods have recently been extended to include more fine-grained decomposition of trait variances, both for phenotypes (for example, multi-trait models^25^) and genotypes (partitioning sites by chromosome^32^, allele frequency^33^ or functional class^34^). In latter group, the genetic covariance matrix is partitioned by allele category, and a BLUP model is fit for each. BLUP is a linear combination of training data, with uncertainties stemming from genetic relatedness only for prediction. Accordingly, we found that genomic BLUP estimates became uncertain when closer relatives were unavailable (Fig. 4a), and prediction error increased. This source of error is not circumvented by the partitioning methods, as the relatedness-derived uncertainty remains, and therefore these approaches are unlikely to improve our suboptimal predictions for more distant relatives.

It is important to note that our study population does not share many of the features of human cohorts. We used data from a diallel cross, in which only two alleles are present at any locus, and their frequencies are close to 50%; there is no spectrum of low frequency and rare alleles. Further, due to the controlled phenotyping design, there is little environmental variation and the heritability estimates in our populations are therefore very high. Although this is atypical for most human traits, our results concern prediction accuracies relative to the heritabilities, regardless of their numerical value. Finally, human complex traits can be influenced by hundreds if not thousands of loci. Nevertheless, their combined predictive ability has remained far below the narrow-sense heritability estimates. We capture nearly all of the broad-sense heritability with the most precise models, demonstrating that knowledge of additional phenotypes helps estimate the combined influence of small effect alleles and interactions that are difficult to map. Therefore, making use of the accumulated personal phenotype data is also expected to improve human trait prediction.

When no very close relatives were available, and no single QTL explained a large fraction of variance, the pure genomic methods were inaccurate, even in our population of 6,642 individuals with high relatedness. At the same time, when the number of very close relatives in the training sample was sufficiently large, the predictions were not improved by adding all remaining more distant relatives. Thus, observing phenotypes for parental haplotypes in at least a few cases causes BLUP to upweight their contributions, and for QTL mapping to prioritize alleles that capture their signal. In concert, these observations suggest that efforts directed towards creating genotype-based scores using common variants to predict disease risk could benefit dramatically from being complemented by systematic collection of family history and relatedness data^30^,^35^,^36^. As information from as few as five close relatives gave large gains, we expect such an approach to be a cost-effective solution for achieving better prediction in a clinical setting with finite resources.

## Methods

### Panel design and phenotyping

172 haploid F_12_ segregants (86 MatA and 86 MatAlpha) from a cross between YPS128 and DVPBG6044 (ref. 37) were crossed in an all against all fashion to obtain 86 × 86=7,396 diploid hybrids using standard yeast protocols (Fig. 1). After removing strains spawning from one contaminated and eight aneuploid haploid founders, we were left with 81 × 82=6,642 crosses for analysis. The strains were grown in biological and technical duplicates (four measurements total) in 1536-position solid agar plate cultures, with all replicates on different plates and taken from two different pre-cultures to reduce systematic bias. Medium preparation, plate pouring, robotic pinning and pre-culture and experimental conditions were all extensively standardized to reduce systematic bias. Every fourth position was occupied by genetically identical internal controls in the form of the reference YPS128 strain, and the 384 controls on each plate were used to remove any remaining bias by normalization. Although complete randomization with respect to all known confounders (for example, plate position, fixture position, machine, pre-culture, temperature, humidity, neighbouring colony size, amount of light) and unknown sources of bias is not feasible, the dense grid of reference strains provides an excellent standard. We extracted the area under the growth curve relative to the starting point in each of the nine environments, converted the values to log-scale, and normalized them to a surface constructed from the surrounding internal YPS128 controls, as described earlier^24^. The four replicate values were then averaged to obtain the final phenotype (that is, mean growth) for each individual and environment. Panel design, genotyping, phenotyping and normalization are described in detail in refs 23, 24.

### Modelling and predictions

We used a range of models to predict a trait of interest either on genomic information only, individual phenotypic information only or both.

*Phenotype (‘P')*. Let *y* be the vector containing the phenotype of interest for all *N* individuals, and let *P*_1_*, …, P*_8_ be the remaining phenotypes. We modelled *y* as 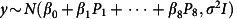 to fit the phenotype weights *β* used for prediction.

*Best linear unbiased predictor*. Let *x*_*j*_ be the genotype vector for SNP *j=*1, …, *M*, and let *X* be the genotype matrix *X=(x*_1_, …, *x*_*M*_). In the genomic BLUP model, 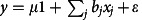 with random coefficients 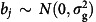 and measurement noise 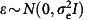. This model implies the multivariate Gaussian distribution 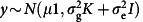, where 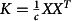 is the realized genetic relatedness matrix, with the scaling constant *c* being the average diagonal value of *XX*^*T*^. Prediction for the test individual can be obtained by conditioning on the observed data in a standard way for multivariate normal distributions. When calculating the standard deviation of the predictive distribution (Fig. 4a), we averaged the variances on the predictive distributions (that is, averaged the diagonal elements of the covariance matrix of the predictive multivariate normal distribution) and reported the square root of this number.

*Quantitative trait loci*. To identify the strongest QTLs, we first carried out forward selection for up to 50 iterations in the linear regression model 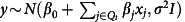, where *Q*_*t*_ denotes the selected collection of QTL indexes at iteration *t*. The number of QTLs in the final model was determined by out-of-sample prediction accuracy, with fourfold cross-validation on the training portion of data (hence, altogether a double cross-validation scheme).

*Midparent*. Let *y*_*ij*_ be the phenotype for individual who has parents *i* and *j*. Let *P*_*i*_^1^ and *P*_*j*_^2^ be the parental phenotype values. We model *y*_*ij*_ as the mid-parent value 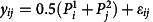, where *ɛ*_*ij*_ is uncorrelated noise. We first fit the parental values from the *y*_*ij*_ observed in training data, and used them to predict phenotypes of test individuals.

*LMM with dominance and interaction effects*. The LMM model combines additive, dominance and interaction effects with genetic relatedness, 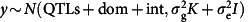. The fixed effects (QTLs+dom+int) are constructed with forward selection among additive QTLs and interaction between all such SNP pairs *x*_*i*_ and *x*_*j*_, where *x*_*i*_ has previously been selected into the model. Although we miss interactions where neither locus has a significant additive effect, it has been shown that such occurrences are rare^38^, and their contribution to explaining variance is negligible^19^. By allowing self-interactions, we also incorporated dominance effects. We selected the final model by performing cross-validation on training data after each of the feature selection steps.

*LMM including phenotypes (‘LMM+P')*. The LMM+P model combines additive, dominance and interaction effects with genetic relatedness and other traits, 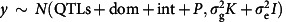. The fixed effects contains a genetic (QTLs+dom+int) and non-genetic (*P*) part. The latter includes the linear combination of all other traits *P*_1_*, …, P*_8_. First, we regress *y* on *P*, and then we construct the genetic component as described for the LMM model.

*Multi-trait LMM*. MT-LMMs model multiple phenotypes jointly. The correlation between two traits is modelled in two parts, via a genetic and non-genetic component as follows^25^. Let *Y*=[*y*_!_,…,*y*_9_] be the matrix for phenotypes *y*_!_,…,*y*_9_, and let *F* denote the fixed effects for each of these phenotypes, *F*=[*f*_1_,…,*f*_9_]. We used the same fixed effects *f*_*i*_ that we constructed in the LMM model. Let *C* be the genetic covariance matrix between phenotypes and Σ the non-genetic one. Then 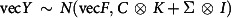 according to the MT-LMM. To obtain MT-LMM predictions which correspond to the LMM+P model, we condition the multivariate normal distribution.

*Mixed random forest*. We applied the MRF approach^28^, available via LIMIX^25^. We ran the MRF with 25 trees and otherwise default settings. For genomic predictions (corresponding to the LMM model), we included all SNPs as potential features. For genomic and phenomic prediction (corresponding to the LMM+P model), we added also other phenotypes as potential features.

### Training and obtaining predictions

All models were fitted with the Python package LIMIX^25^. We used four-fold cross-validation to obtain out-of-sample predictions for all 6642 individuals. We partitioned the set of all individuals into four folds analogously as shown in Fig 3a, i.e. by splitting the two sets of parents (i.e. one in rows, the other in columns) into two equally sized groups. We use each one of these four subsets of size *N*^2^ as a test set to obtain predictions and the remaining three as a training set to fit the models. First, we did not take into account the relatedness structure and divided individuals into subsets randomly (results in Fig. 2). Later, we distinguished between closely and distantly related individuals (results in Fig. 3). The latter correspond to siblings in a traditional sense, sharing many of the haplotype blocks (expected fraction of sites identical by state 0.375), whereas the former share one complete chromosome in each pair (expected fraction of sites identical by state 0.5). The four test sets remained the same as before, but instead of training on all 3*N*^2^ remaining individuals, we picked the *N* × *N* individuals who do not share a parent with anyone in the test set (‘distant relatives'), as well as sampled *N*^2^ from the 2*N*^2^ remaining individuals who do share one parent with someone in the test set (‘close relatives').

### Heritability estimation

Narrow-sense heritability was estimated from the genomic BLUP model as 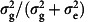, when fitted to all of the data. To estimate repeatability, we fitted the following fixed effects model *r*_*ij*_=*y*_*i*_+*ɛ*_*ij*_, where *r*_*i1*_*, r*_*i2*_*, r*_*i3*_*, r*_*i4*_ are the four replicate measurements for individual *i*, *y*_*i*_ is the average *r*_*ij*_ value for this individual and *ɛ*_*ij*_∼*N*(0, *σ*^2^). Repeatability was estimated as 1−*σ*^2^/Var(*r*).

### Data availability

The data used in this study are available in the Supporting Information of Hallin *et al*.^23^ Analysis code is available at https://github.com/kasparmartens/y10k-prediction.

## Additional information

**Code availability:** All the code used in the current study is available at https://github.com/kasparmartens/y10k-prediction.

**How to cite this article:** Märtens, K. *et al*. Predicting quantitative traits from genome and phenome with near perfect accuracy. *Nat. Commun.* 7:11512 doi: 10.1038/ncomms11512 (2016).

## Supplementary Material

Supplementary Information Supplementary Figures 1-10, Supplementary Tables 1-3 and Supplementary References
