# Supplementary material for: Predicting quantitative traits from genome and phenome with near perfect accuracy
Source: Nat Commun. 2016 May 10;7:11512. doi: 10.1038/ncomms11512 (PMC4866306; doi:10.1038/ncomms11512)
Supplement: Supplementary Information — Supplementary Figures 1-10, Supplementary Tables 1-3 and Supplementary References [file ncomms11512-s1.pdf]

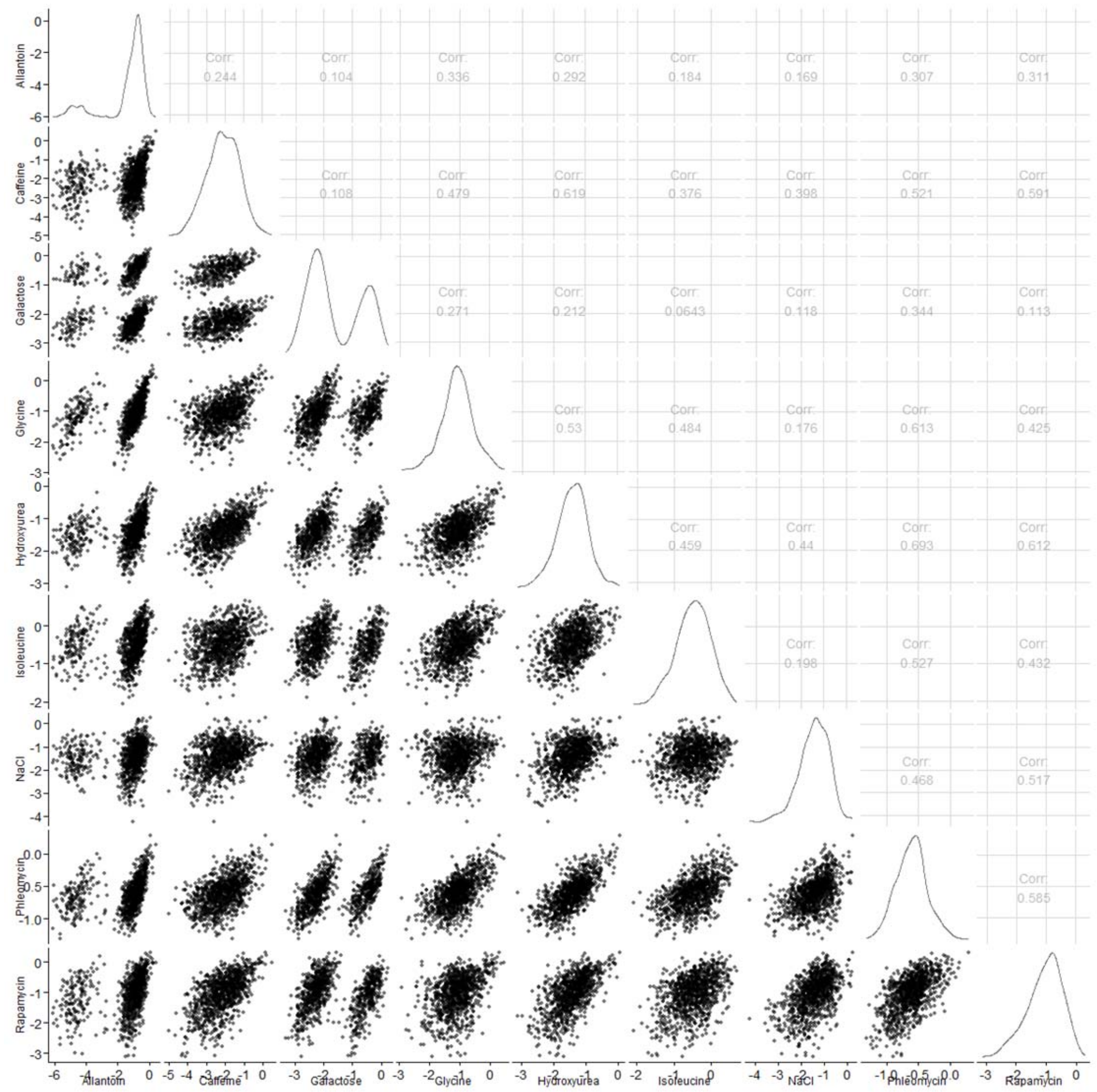

Supplementary Figure 1. Pairwise associations between traits.

Pairwise scatterplots of the traits (lower triangle, based on a random subsample of 1000 points), together with the Pearson correlation coefficient (upper triangle).

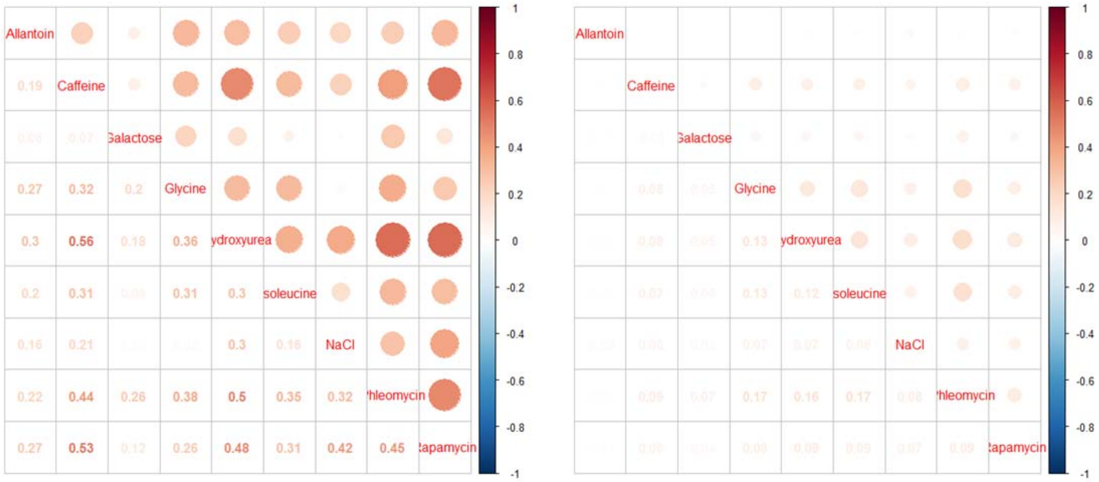

Supplementary Figure 2. Covariance between phenotypes decomposed into genetic (left) and non-genetic (right) components. These values were obtained from fitting a multi-trait linear mixed model to all phenotypes simultaneously.

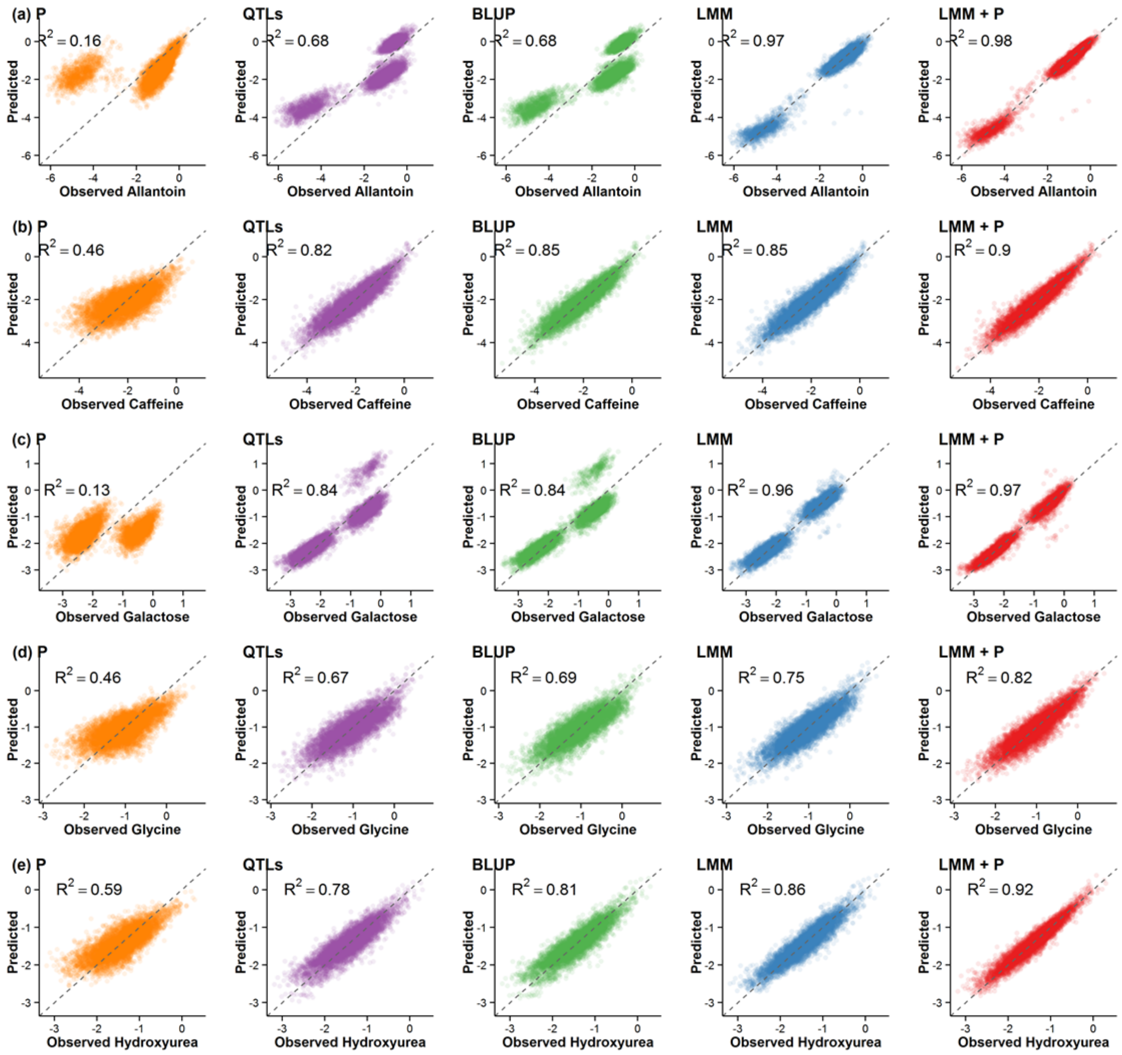

(a) P
R² = 0.16
QTLs
R² = 0.68
BLUP
R² = 0.68
LMM
R² = 0.97
LMM + P
R² = 0.98
Predicted
Observed Allantoin
(b) P
R² = 0.46
QTLs
R² = 0.82
BLUP
R² = 0.85
LMM
R² = 0.85
LMM + P
R² = 0.9
Predicted
Observed Caffeine
(c) P
R² = 0.13
QTLs
R² = 0.84
BLUP
R² = 0.84
LMM
R² = 0.96
LMM + P
R² = 0.97
Predicted
Observed Galactose
(d) P
R² = 0.46
QTLs
R² = 0.67
BLUP
R² = 0.69
LMM
R² = 0.75
LMM + P
R² = 0.82
Predicted
Observed Glycine
(e) P
R² = 0.59
QTLs
R² = 0.78
BLUP
R² = 0.81
LMM
R² = 0.86
LMM + P
R² = 0.92
Predicted
Observed Hydroxyurea

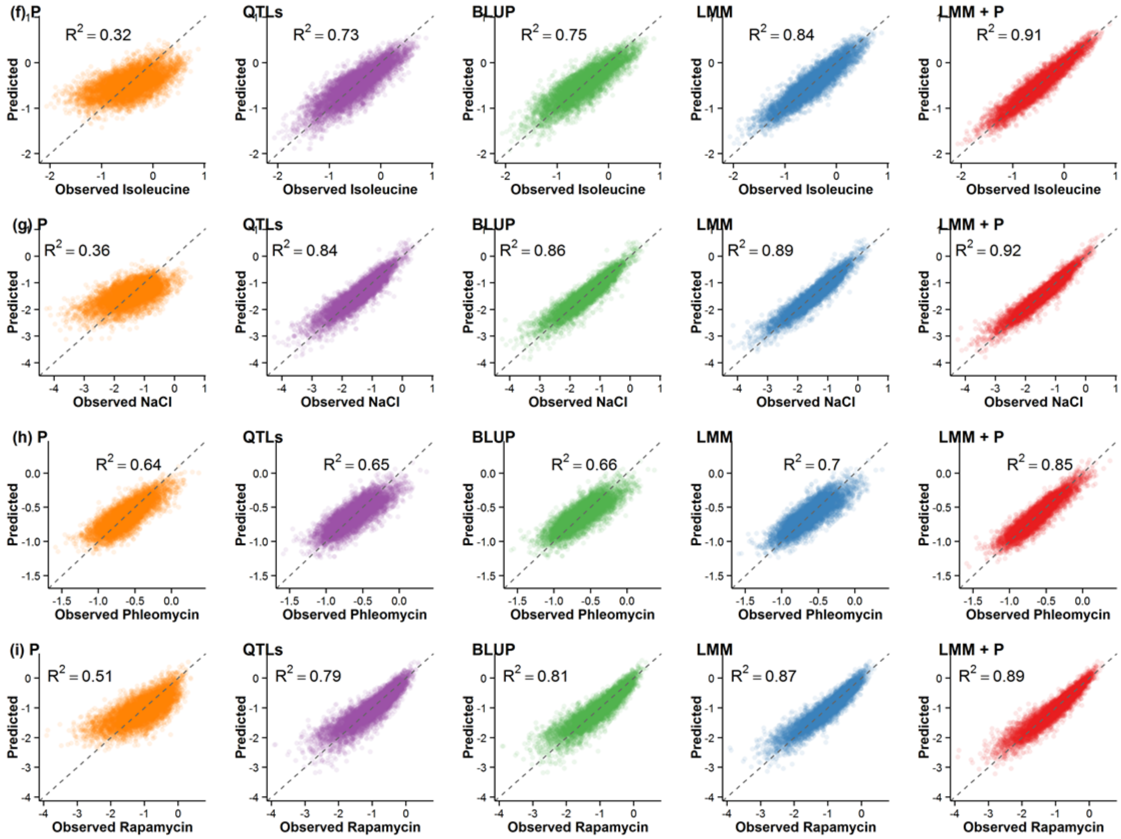

Supplementary Figure 3. Prediction quality for all environments.
As in Figure 2a, predicted (y-axis) and observed (x-axis) growth is shown for four model classes, but now for all nine environments: (a) allantoin, (b) caffeine, (c) galactose, (d) glycine, (e) hydroxyurea, (f) isoleucine, (g) NaCl, (h) phleomycin, (i) rapamycin.

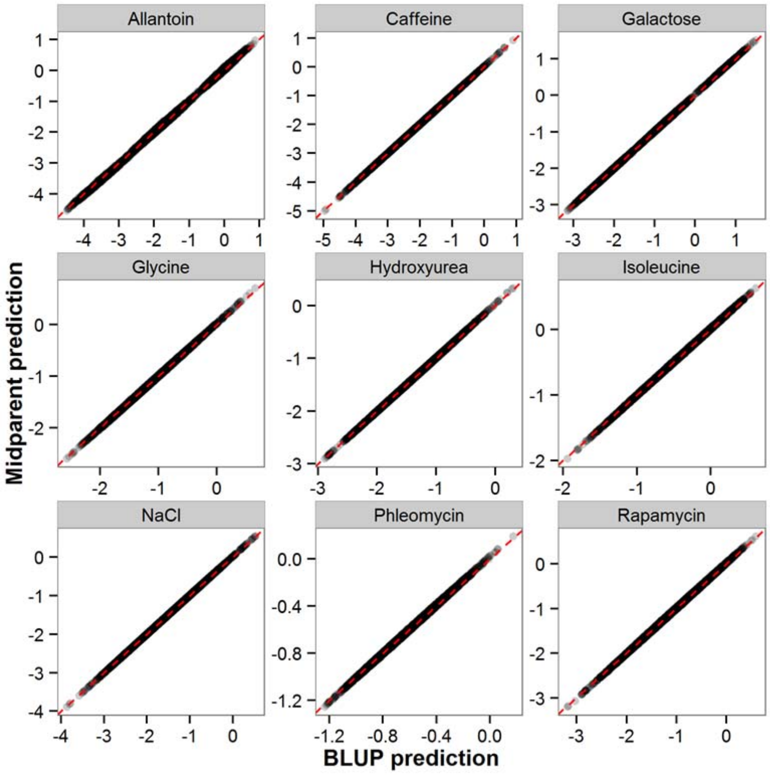

Supplementary Figure 4. Predictions from the genomic BLUP model (x-axis) are almost identical to the predictions from the midparent model (y-axis) for all nine environments. The red dashed line shows the identity y=x. All predictions were obtained by four-fold cross-validation.

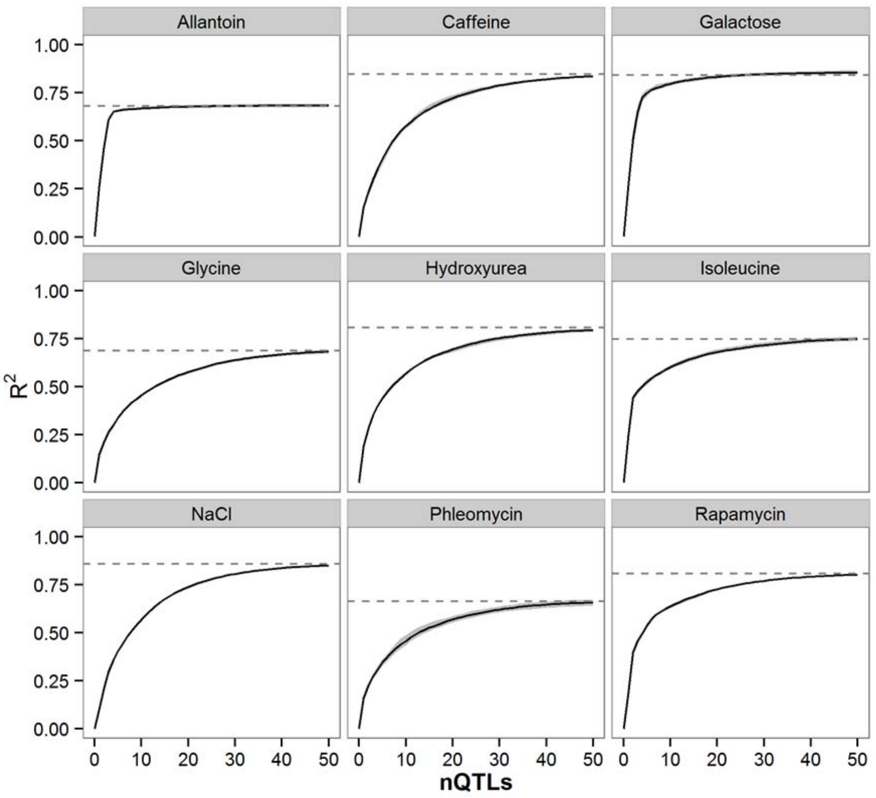

Supplementary Figure 5. With a large number of QTLs, the QTL based model performs almost as well as BLUP. The R^2 of the QTL model (y-axis) for various number of QTLs included (x-axis), connected with a black line. The grey dashed horizontal line shows the prediction accuracy of BLUP.

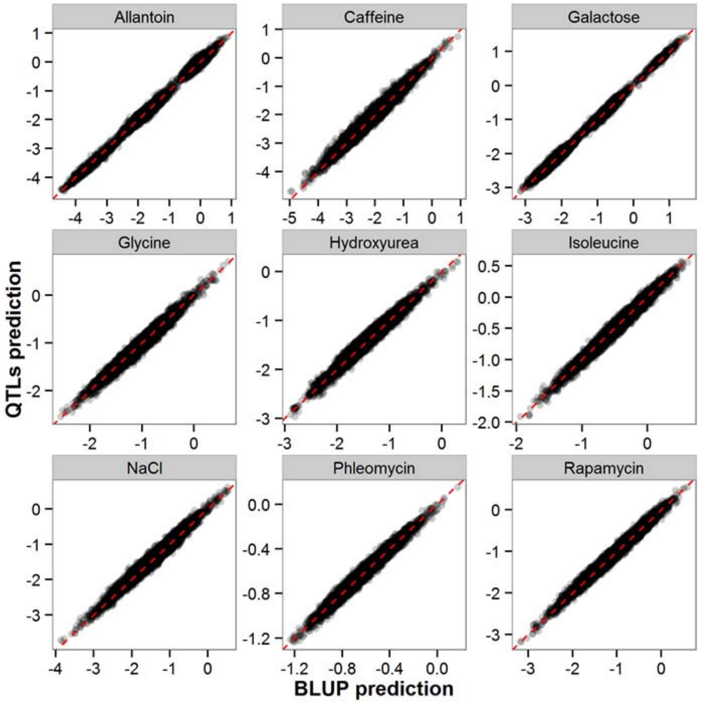

Supplementary Figure 6. The BLUP predictions (x-axis) are highly similar to the QTL model predictions (y-axis) for all nine environments. The red dashed line shows the identity y=x. All predictions were obtained by four-fold cross-validation.

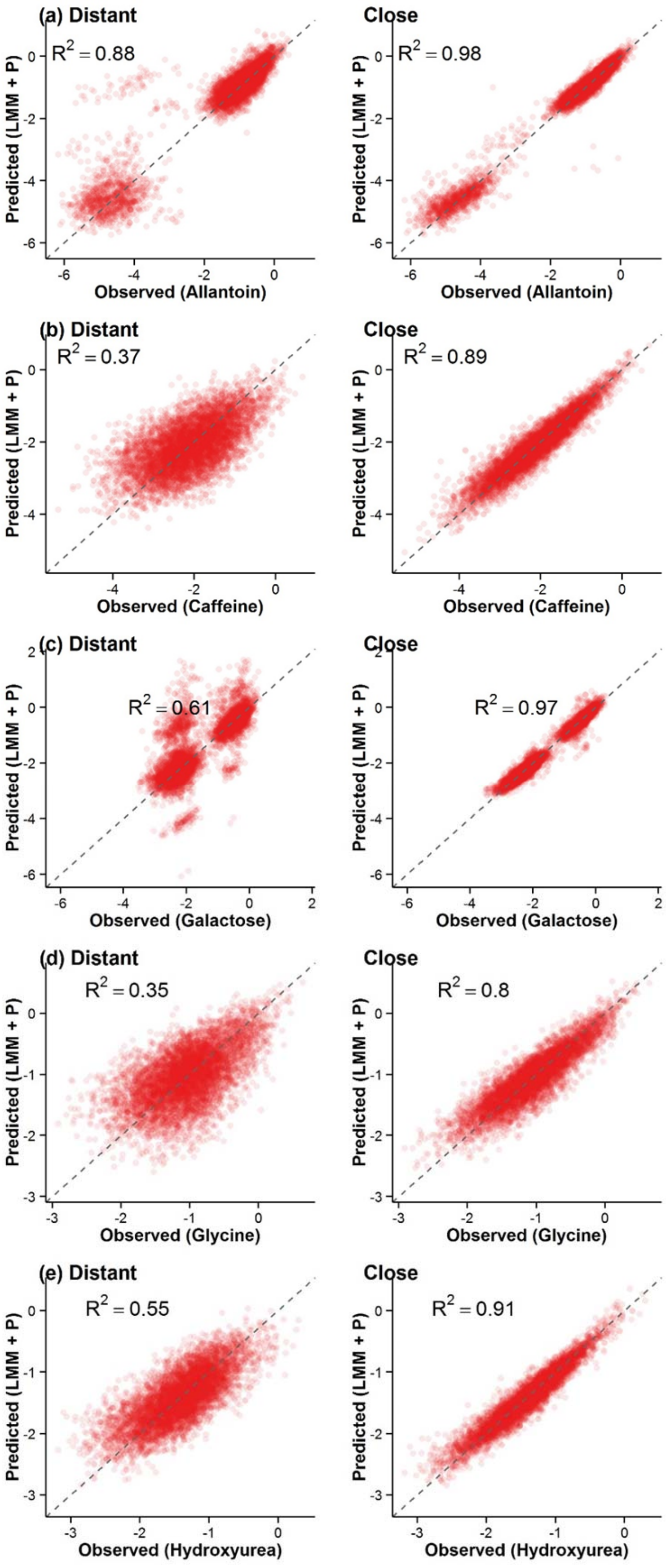
(a) Distant
R² = 0.88
Close
R² = 0.98
Predicted (LMM + P)
Observed (Allantoin)
(b) Distant
R² = 0.37
Close
R² = 0.89
Observed (Caffeine)
(c) Distant
R² = 0.61
Close
R² = 0.97
Observed (Galactose)
(d) Distant
R² = 0.35
Close
R² = 0.8
Observed (Glycine)
(e) Distant
R² = 0.55
Close
R² = 0.91
Observed (Hydroxyurea)

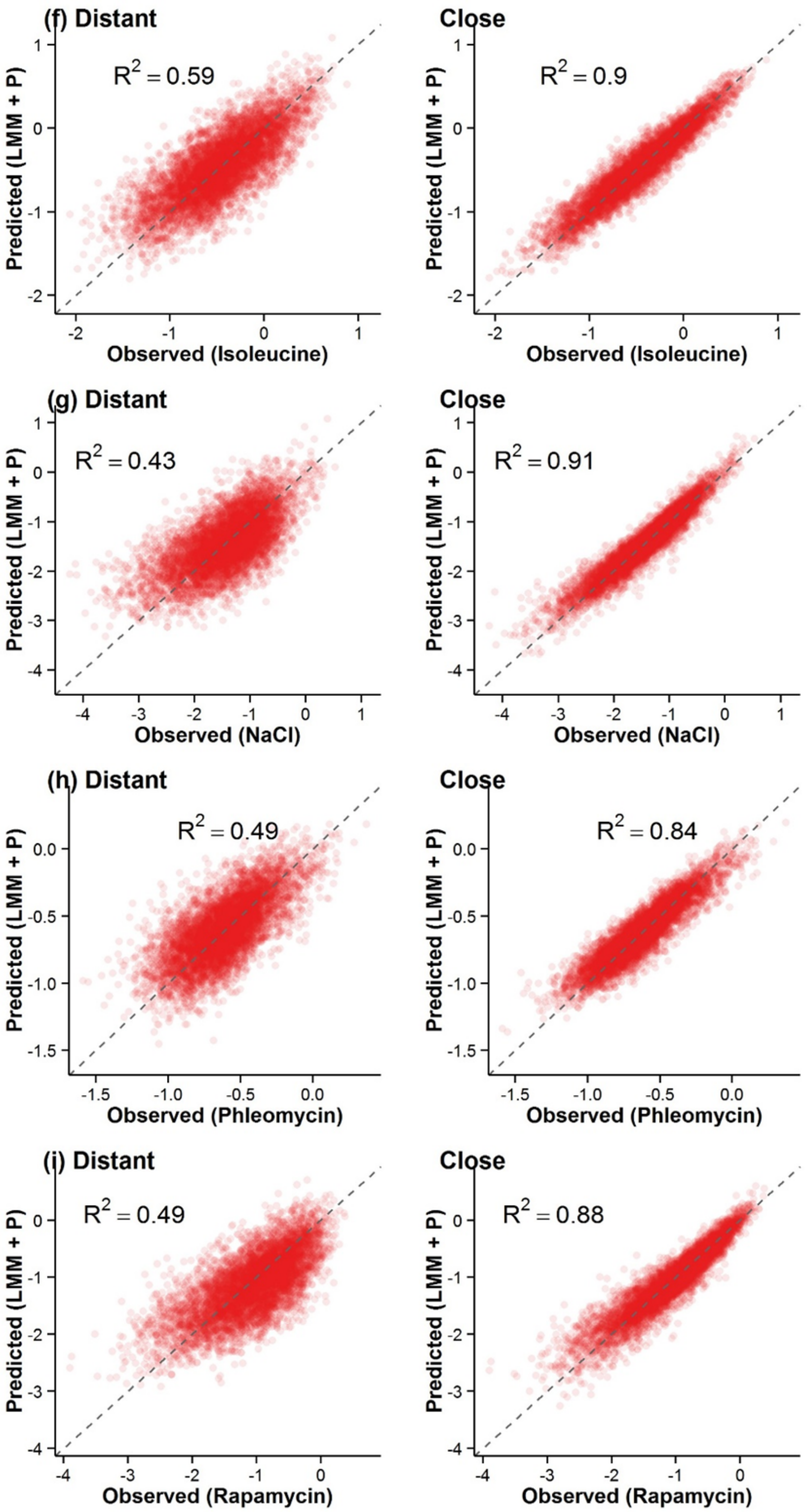

Supplementary Figure 7. As in Figure 3b, predicted (y-axis) and observed (x-axis) growth using the best LMM+P model in different training scenarios, but now for all nine environments: (a) allantoin, (b) caffeine, (c) galactose, (d) glycine, (e) hydroxyurea, (f) isoleucine, (g) NaCl, (h) phleomycin, (i) rapamycin.

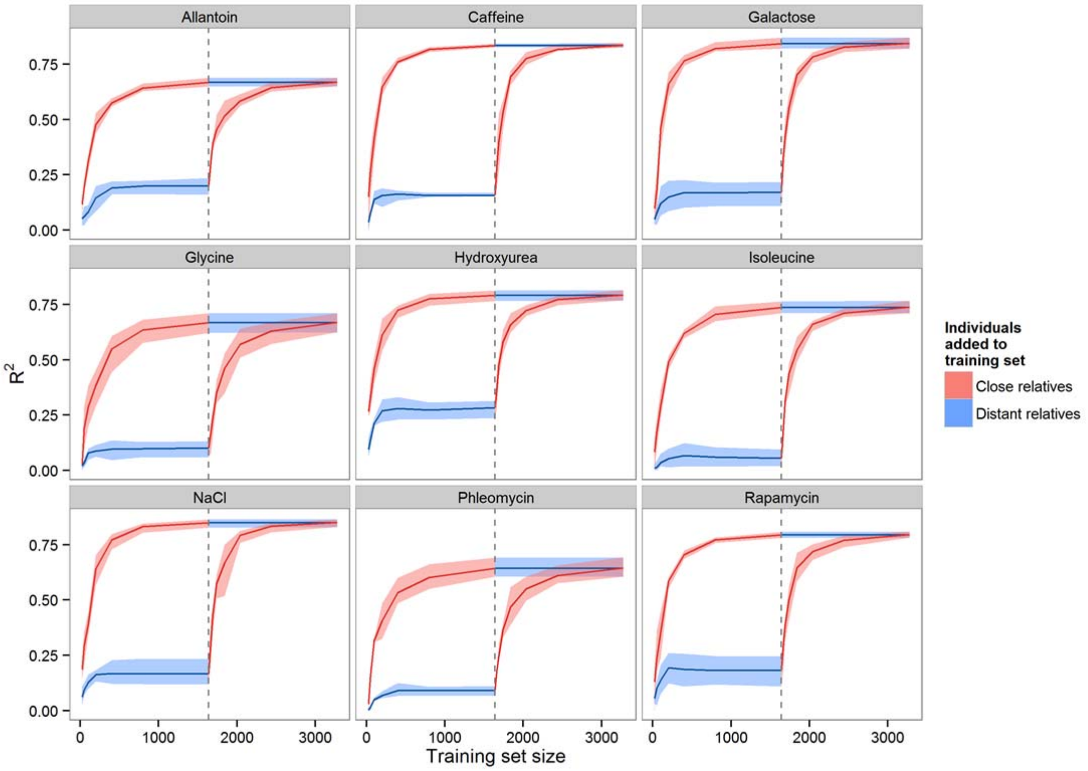

Supplementary Figure 8. As in Figure 4b, predictive performance of BLUP (R^2, y-axis) when expanding the training set (size on x-axis) with individuals closely (red line) or distantly (blue line) related to the test set, but now for all nine environments. From the dashed grey line onwards, distant relatives are added to the training set of closely related individuals, and vice versa. Shaded regions denote the range of R^2 over the four cross-validation folds.

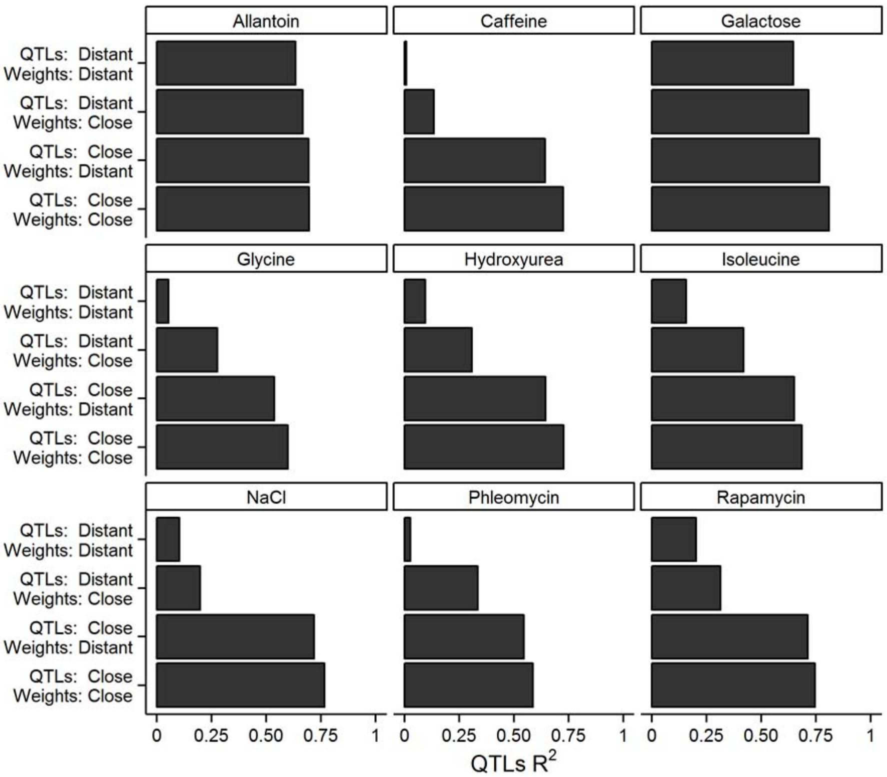

Supplementary Figure 9. As in Figure 4d, predictive performance (R^2, x-axis) of the QTLs model, stratified by training sets used for QTL mapping (model selection) and weight estimation (model fitting), but now for all nine environments. QTL mapping and weight estimation are carried out under four training scenarios (y-axis): both stages in distant relatives ("QTLs: Distant, Weights: Distant"), both in close relatives ("QTLs: Close, Weights: Close"), QTLs mapped in distant relatives and weights estimated in close relatives ("QTLs: Distant, Weights: Close"), or vice versa ("QTLs: Distant, Weights: Close").

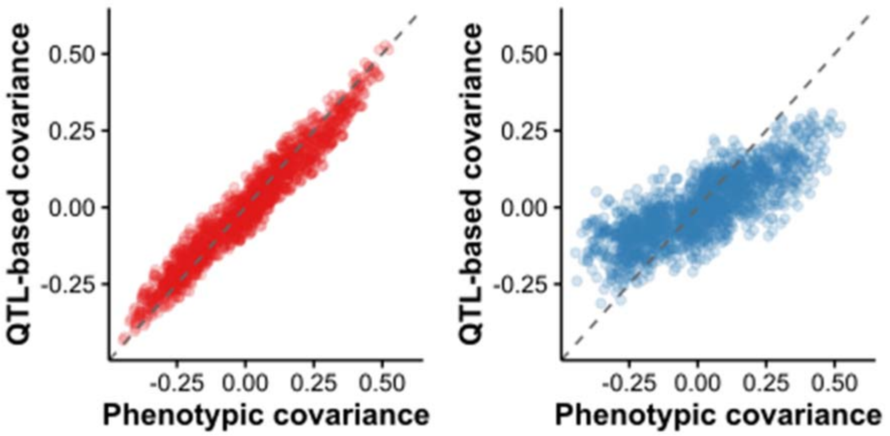

Supplementary Figure 10. QTL model approximates phenotypic rather than genetic covariance, and this estimated correlation structure does not generalize well for distant relatives. Covariance from QTLs model (*y*-axis) compared to phenotypic (left panel) and genetic (right) covariance, when trained on distant (blue dots) or close relatives (red dots). Grey dashed line denotes the identity *y*=*x*.

Supplementary Table 1. Narrow-sense heritability estimates in nine environments and their standard errors.

	Allantoin	*Caffeine*	*Galactose*	*Glycine*	*Hydroxyurea*	*Isoleucine*	*NaCl*	*Phleo-mycin*	*Rapa-mycin*
h^2	0.656	0.868	0.866	0.731	0.8	0.791	0.885	0.691	0.832
SE	0.075	0.097	0.097	0.083	0.09	0.089	0.099	0.079	0.094

Supplementary Table 2. Repeatability (broad sense heritability) estimates for the average of 1, 2, 3, and 4 replicate measurements

Measure-ments	*Allan-toin*	*Caffeine*	*Galac-tose*	*Glycine*	*Hydroxy-urea*	*Iso-leucine*	*NaCl*	*Phleo-mycin*	*Rapa-mycin*
1	0.961	0.606	0.965	0.747	0.788	0.84	0.908	0.743	0.785
2	0.981	0.789	0.984	0.864	0.894	0.917	0.949	0.862	0.866
3	0.987	0.851	0.989	0.895	0.921	0.94	0.966	0.897	0.917
4	0.99	0.891	0.992	0.918	0.939	0.953	0.974	0.921	0.934

Supplementary Table 3. Detailed overview of the nine environments and their effects on the cell.

**Challenge**	**Concentration**	**Type**	**Cellular effect**
**NaCl**	0.5M	Cation stress	Extracellular Na^+ exposes yeast to hyperosmosis, and requires intracellular glycerol accumulation and cell wall and cytoskeleton strengthening[1]. Na^+ enters cells through the K^+ transporters Trk1 and Trk2, and potentially through Pho89 and Nsc1. Intracellularly, it displaces K^+, challenging cell volume regulation, intracellular pH and membrane potentials, protein synthesis, and enzyme activation[2]. At acidic pH, Na^+ is exported by Nha1[3]. At higher pH, Na^+ efflux is mediated by the Ena proteins, encoded in a single gene in most natural strains but in Wine/European strains a different gene variant introgressed from *S. paradoxus* has been amplified into 3-5 similar paralogs[4]. This introgression/duplication largely defines natural yeast variation in salt tolerance. The Ena1 variant plays the

			critical role in Na^+ tolerance[5]. Regulation of salt tolerance genes is complex, involving the *HOG1*, Calcineurin pathway, *TOR* pathway, *RIM101* and glucose repression pathways.
**Galactose**	2%	Carbon source (replaces glucose)	Galactose well supports yeast growth as only energy and carbon source. It enters cells through the Gal2 permease in a process that also requires Gal1. Intracellular galactose is converted into glucose-1-phosphate by three sequential reactions that are catalyzed by Gal10, Gal1 and Gal7 respectively, with Gal10 also being required for re-cycling of a pathway intermediate. All the galactose structural genes are coordinately regulated at the level of transcription in response to galactose by Gal4p, Gal80p, and Gal3p[6,7]. All seven GAL genes are spatially co-localized in a gene cluster[8]. Natural variation in galactose growth is largely accounted for by loss-of-function mutations in Gal2, Gal3[4] or loss of the whole GAL cluster[8].
**Caffeine**	5mg/mL	Toxin	A purine, similar to adenine and guanine. Binds to multiple, very diverse enzymatic targets. In yeast, caffeine targets the TORC1 complex[9], caffeine prevents gene conversion by displacing Rad51[10], impedes DNA repair[11], and may interfere with cell wall generation. Mutations in diverse cellular components modulate caffeine sensitivity. Caffeine trafficking is not well understood.
**Rapamycin**	24ng/mL	Toxin	A polyketide macrolide produced by *Streptomyces hygroscopicus*. Rapamycin binds the Fpr1 protein[12]. This protein-drug complex binds to and inhibits Tor1[13], but not Tor2[14]. Tor1p is a component of TORC1, a protein complex regulating nutrient availability and stress responses[15]. Mutations in Fpr1 or Tor1 confer resistance to the drug[13]. Loss of non-essential TORC1 components (e.g. Kog1 and Tco089), or in TORC1 interactors (e.g. Rrd1) confer sensitivity. Rapamycin binds to the Fpr1 paralog Fpr2 without known toxic effects[16]. Rapamycin trafficking is not well understood.

**Phleomycin**	2μ/mL	Toxin	A 12 component drug complex isolated from *Streptomyces*. Binds to DNA, impedes DNA polymerase and induces DNA damage and breakdown, arresting cells[17]. Loss of DNA repair components, such as Rad6, 9 or 17 cause phleomycin hypersensitivity[18,19]. Phleomycin trafficking is not well understood.
**Hydroxyurea**	2.5mg/mL	Toxin	Hydroxyurea inhibits reduction of ribonucleotides to deoxidized ribonucleotides, by binding to and inhibiting the four component RNR (ribonucleotide reductase) complex [20]. dNTP depletion impedes synthesis and repair of DNA[2122], arresting cells in early S-phase. The RNR complex consists of four proteins, *RNR1*, *2*, *3*, and *4*, of which *RNR1* and *2* are essential, and *RNR4*'s essentiality depends on the genetic background[23,24]. Natural variation in hydroxyurea resistance is partially mediated via the Hur1 protein[4].
**Glycine**	30mg N/L	Nitrogen source (replaces ammonium)	Glycine is generally a very poor nitrogen source for yeast, but the growth delays, rates and efficiencies vary between strains[25]. Glycine has no dedicated high affinity permease but is taken up by the broad specificity amino acid permeases Gap1[26] and Agp1 and the more specialized Dip5 and Put4[27]. Intracellular glycine is catabolized into ammonium in three sequential mitochondrial reactions. These are catalyzed by a single glycine cleavage complex with four components: Gcv3, Gcv1, Gcv2 and Lpd1. Cleavage reactions requires a folic acid derivative.
**Isoleucine**	30mg N/L	Nitrogen source (replaces ammonium)	Isoleucine is generally a poor nitrogen source for yeast, but growth delays, rates and efficiencies vary between strains[25]. Isoleucine is take up by the paralogous high affinity permeases Bap2[28] and Bap3[27] as well the low affinity general amino acid permease Gap1[26]. Isoleucine is catabolized in a single step reaction to glutamate, using the mitochondrial Bat1 or the cytoplasmic Bat2[29], with Bat1 expressed during exponential growth and Bat2 in stationary phase.
**Allantoin**	30mg N/L	Nitrogen source (replaces	Allantoin is the primary nitrogen secretion product of higher mammals,

	ammonium)	excluding apes. Yeast utilizes allantoin as sole nitrogen source, but with varying delays, rates and efficiencies that largely maps to variation in Dal4 and Dal1[25]. The Dal4 permease is the only known entrance mechanism for allantoin. Intracellular allantoin is catabolized to urea in three sequential reactions catalyzed by Dal1, Dal2 and Dal3 respectively[30-32]. Urea is converted into ammonium by the multi-step enzyme Dur1,2[33]. The allantoin catabolic genes are regulated by both general and specific signals that involve Gln3, Gat1, Dal80, Dal81, and Dal82[34]. The *DAL* genes are encoded in a tight gene cluster on Chr IX[35].

Supplementary references

1. Hohmann, S. Osmotic stress signaling and osmoadaptation in yeasts. *Microbiol Mol Biol Rev* **66**, 300-72. (2002).
2. Arino, J., Ramos, J. & Sychrova, H. Alkali metal cation transport and homeostasis in yeasts. *Microbiol Mol Biol Rev* **74**, 95-120 (2010).
3. Banuelos, M.A., Sychrova, H., Bleykasten-Grosshans, C., Souciet, J.L. & Potier, S. The Nha1 antiporter of Saccharomyces cerevisiae mediates sodium and potassium efflux. *Microbiology* **144 (Pt 10)**, 2749-58 (1998).
4. Warringer, J. *et al.* Trait variation in yeast is defined by population history. *PLoS Genet* **7**, e1002111 (2011).
5. Haro, R., Garciadeblas, B. & Rodriguez-Navarro, A. A novel P-type ATPase from yeast involved in sodium transport. *FEBS Lett* **291**, 189-91 (1991).
6. Johnston, M. & Davis, R.W. Sequences that regulate the divergent GAL1-GAL10 promoter in Saccharomyces cerevisiae. *Mol Cell Biol* **4**, 1440-8 (1984).
7. Lohr, D., Venkov, P. & Zlatanova, J. Transcriptional regulation in the yeast GAL gene family: a complex genetic network. *Faseb J* **9**, 777-87 (1995).
8. Slot, J.C. & Rokas, A. Multiple GAL pathway gene clusters evolved independently and by different mechanisms in fungi. *Proc Natl Acad Sci U S A* **107**, 10136-41 (2010).
9. Reinke, A., Chen, J.C., Aronova, S. & Powers, T. Caffeine targets TOR complex I and provides evidence for a regulatory link between the FRB and kinase domains of Tor1p. *J Biol Chem* **281**, 31616-26 (2006).
10. Tsabar, M., Mason, J.M., Chan, Y.L., Bishop, D.K. & Haber, J.E. Caffeine inhibits gene conversion by displacing Rad51 from ssDNA. *Nucleic Acids Res* **43**, 6902-18 (2015).
11. Tsabar, M. *et al.* Caffeine impairs resection during DNA break repair by reducing the levels of nucleases Sae2 and Dna2. *Nucleic Acids Res* **43**, 6889-901 (2015).
12. Koltin, Y. *et al.* Rapamycin sensitivity in Saccharomyces cerevisiae is mediated by a peptidyl-prolyl cis-trans isomerase related to human FK506-binding protein. *Mol Cell Biol* **11**, 1718-23 (1991).
13. Heitman, J., Movva, N.R. & Hall, M.N. Targets for cell cycle arrest by the immunosuppressant rapamycin in yeast. *Science* **253**, 905-9 (1991).
14. Loewith, R. *et al.* Two TOR complexes, only one of which is rapamycin sensitive, have distinct roles in cell growth control. *Mol Cell* **10**, 457-68 (2002).

15. Loewith, R. & Hall, M.N. Target of rapamycin (TOR) in nutrient signaling and growth control. *Genetics* **189**, 1177-201 (2011).
16. Nielsen, J.B. *et al.* Yeast FKBP-13 is a membrane-associated FK506-binding protein encoded by the nonessential gene FKB2. *Proc Natl Acad Sci U S A* **89**, 7471-5 (1992).
17. Nakada, D., Shimomura, T., Matsumoto, K. & Sugimoto, K. The ATM-related Tel1 protein of Saccharomyces cerevisiae controls a checkpoint response following phleomycin treatment. *Nucleic Acids Res* **31**, 1715-24 (2003).
18. Belenguer, P., Oustrin, M.L., Tiraby, G. & Ducommun, B. Effects of phleomycin-induced DNA damage on the fission yeast Schizosaccharomyces pombe cell cycle. *Yeast* **11**, 225-31 (1995).
19. He, C.H., Masson, J.Y. & Ramotar, D. A Saccharomyces cerevisiae phleomycin-sensitive mutant, ph140, is defective in the RAD6 DNA repair gene. *Can J Microbiol* **42**, 1263-6 (1996).
20. Krakoff, I.H., Brown, N.C. & Reichard, P. Inhibition of ribonucleoside diphosphate reductase by hydroxyurea. *Cancer Res* **28**, 1559-65 (1968).
21. Yao, R. *et al.* Subcellular localization of yeast ribonucleotide reductase regulated by the DNA replication and damage checkpoint pathways. *Proc Natl Acad Sci U S A* **100**, 6628-33 (2003).
22. Koc, A., Wheeler, L.J., Mathews, C.K. & Merrill, G.F. Hydroxyurea arrests DNA replication by a mechanism that preserves basal dNTP pools. *J Biol Chem* **279**, 223-30 (2004).
23. Elledge, S.J. & Davis, R.W. Two genes differentially regulated in the cell cycle and by DNA-damaging agents encode alternative regulatory subunits of ribonucleotide reductase. *Genes Dev* **4**, 740-51 (1990).
24. Huang, M. & Elledge, S.J. Identification of RNR4, encoding a second essential small subunit of ribonucleotide reductase in Saccharomyces cerevisiae. *Mol Cell Biol* **17**, 6105-13 (1997).
25. Ibstedt, S. *et al.* Concerted evolution of life stage performances signals recent selection on yeast nitrogen use. *Mol Biol Evol* **32**, 153-61 (2015).
26. Jauniaux, J.C. & Grenson, M. GAP1, the general amino acid permease gene of Saccharomyces cerevisiae. Nucleotide sequence, protein similarity with the other bakers yeast amino acid permeases, and nitrogen catabolite repression. *Eur J Biochem* **190**, 39-44 (1990).
27. Regenberg, B., During-Olsen, L., Kielland-Brandt, M.C. & Holmberg, S. Substrate specificity and gene expression of the amino-acid permeases in Saccharomyces cerevisiae. *Curr Genet* **36**, 317-28 (1999).
28. Grauslund, M., Didion, T., Kielland-Brandt, M.C. & Andersen, H.A. BAP2, a gene encoding a permease for branched-chain amino acids in Saccharomyces cerevisiae. *Biochim Biophys Acta* **1269**, 275-80 (1995).
29. Kispal, G., Steiner, H., Court, D.A., Rolinski, B. & Lill, R. Mitochondrial and cytosolic branched-chain amino acid transaminases from yeast, homologs of the myc oncogene-regulated Eca39 protein. *J Biol Chem* **271**, 24458-64 (1996).
30. Buckholz, R.G. & Cooper, T.G. The allantoinase (DAL1) gene of Saccharomyces cerevisiae. *Yeast* **7**, 913-23 (1991).
31. Yoo, H.S. & Cooper, T.G. Sequences of two adjacent genes, one (DAL2) encoding allantoicase and another (DCG1) sensitive to nitrogen-catabolite repression in Saccharomyces cerevisiae. *Gene* **104**, 55-62 (1991).
32. Yoo, H.S. & Cooper, T.G. The ureidoglycollate hydrolase (DAL3) gene in Saccharomyces cerevisiae. *Yeast* **7**, 693-8 (1991).
33. Cooper, T.G., Lam, C. & Turoscy, V. Structural analysis of the dur loci in S. cerevisiae: two domains of a single multifunctional gene. *Genetics* **94**, 555-80 (1980).
34. Magasanik, B. & Kaiser, C.A. Nitrogen regulation in Saccharomyces cerevisiae. *Gene* **290**, 1-18 (2002).
35. Naseeb, S. & Delneri, D. Impact of chromosomal inversions on the yeast DAL cluster. *PLoS One* **7**, e42022 (2012).
